# Adenoma of the nonpigmented ciliary epithelium presenting as glaucoma

**DOI:** 10.1016/j.ajoc.2023.101871

**Published:** 2023-06-20

**Authors:** Gustav Stålhammar, Bertil E. Damato, Maria Fili

**Affiliations:** a St. Erik Eye Hospital, Stockholm, Sweden; b Department of Clinical Neuroscience, Division of Ophthalmology and Vision, Unit of Ocular Oncology and Pathology, Karolinska Institutet, Stockholm, Sweden

## Abstract

**Purpose:**

We describe a case of adenoma of the nonpigmented ciliary epithelium in a 58-year-old male, who presented with glaucoma.

**Observations:**

A healthy White male was incidentally found to have an elevated intraocular pressure in his left eye (25 mmHg) during a visit to a local optometrist. After further investigations he was diagnosed with a primary open angle glaucoma (POAG) and treated with drops for two years until he developed a sectorial cataract. During the first dilated eye exam, a pale tan tumor was discovered, that seemed to originate from the superior ciliary body, causing a sectorial-cortical cataract and subluxation of the lens. The eye was enucleated on the suspicion of a rare adult medulloepithelioma, because of multicystic features on B-scan ultrasonography. However, histopathological examination revealed an adenoma of the nonpigmented ciliary epithelium that grew in trabecular papillary patterns, with smaller areas of solid and microcystoid growth. As this is a benign tumor without metastatic potential, the patient was referred back to his home clinic without requirement for radiological staging or screening.

**Conclusion and Importance:**

Adenomas of the nonpigmented ciliary epithelium (NPCE adenomas) are benign tumors that are often mistaken for malignant counterparts. Thus, this case report expands on the available literature of this rare entity.

## 1 Introduction

Adenomas of the non-pigmented epithelium of the ciliary body (NPCEA) are rare, benign neoplasms, with less than 40 cases reported in the English literature.^1^, ^2^, ^3^, ^4^, ^5^, ^6^, ^7^, ^8^, ^9^, ^10^, ^11^, ^12^, ^13^, ^14^, ^15^, ^16^, ^17^, ^18^ NPCEA have been associated with elevated levels of vascular endothelial growth factor (VEGF) in vitreous and aqueous humor. Co-morbidities include rubeosis iridis, secondary glaucoma, vitreous hemorrhage, optic disc neovascularization and macular edema.^4^^,^^14^^,^^17^^,^^18^ Originating in the ciliary body, NPCEA frequently cause secondary cataract and subluxation of the lens.^1^^,^^4^^,^^6^

In most previous reports, NPCEA have been diagnosed clinically as a ciliary body melanoma, and the correct diagnosis was not made until the eye was examined histopathologically after enucleation.^13^ In this case, we present a 58-year old male who was referred to us with a pale tan ciliary body tumor that was discovered during cataract surgery planning, after having been unsuccessfully treated for an elevated intraocular pressure (IOP) for two years.

## 2 Case report

In August 2020, a 55-year-old, healthy and asymptomatic White male underwent an examination by an optometrist in southern Sweden to renew his eyeglass prescription. He used no medications and had had very few interactions with health care providers, most notably a laser-assisted in-situ keratomileusis (LASIK) ten years prior. His best-corrected visual acuity (BCVA) was 20/20 in each eye. Routine air-puff tonometry showed the IOP in his left eye to be elevated to 25 mmHg, while the IOP in his right eye was normal (13 mmHg). Therefore, he was referred to a local ophthalmologist who confirmed the pressure difference and ordered an optical coherence tomography (OCT) of the optic nerve head and a 24-2 Humphrey visual field test. Whereas no abnormalities were found in the visual field test, optic disc cupping was noted, and the juxtapapillary retinal nerve fiber layer (RNFL) was thinned in his left eye. No rubeosis iridis or signs of uveitis were noted. When these results were confirmed in renewed visual field testing and OCT 6 months later, the patient was diagnosed with primary open angle glaucoma (POAG) and prescribed timolol 1 mg/ml, once per day in both eyes.

During the following two years, the IOP in his right eye varied between 12 and 18 mmHg, and in his left eye between 13 and 19 mmHg (corrected for central corneal thickness, Supplementary Fig. 1A). The home clinic ophthalmologist replaced timolol with tafluprost 15 μg/ml, and then a combination of timolol and tafluprost in unsuccessful attempts to reach target pressures of approximately 12–13 mmHg. 24-2 Humphrey visual fields remained normal, but the optic disc cupping increased (Supplementary Fig. 1B).

In October 2022, the patient complained of reduced visual acuity in his left eye. The BCVA had now fallen to 20/32, while it was retained at 20/20 in his right eye. In slit lamp biomicroscopy, a cortical cataract was confirmed. He was therefore referred for a cataract surgery, with some hope that this would also reduce IOP slightly.

In November 2022, the now 58-year-old man was seen by the cataract surgeon and underwent his first examination with dilated pupils. A pale tumor with dilated vessels on its surface was discovered superiorly between 11 and 1 o'clock. A small segment of the iris pigment epithelium appeared to be detached. The *retro*-iridial lesion could not be captured on anterior segment OCT (Supplementary Fig. 1C). The plans for cataract surgery were halted and the patient was referred to our ocular oncology service at St. Erik Eye Hospital, Stockholm, on the suspicion of a ciliary body malignancy.

**Fig. 1:**
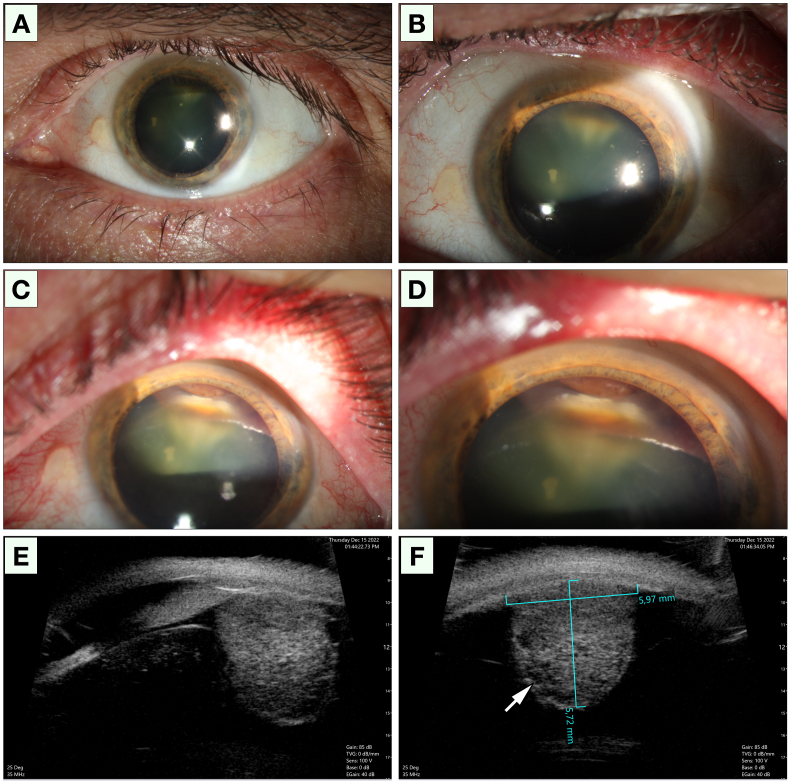
Clinical appearance. A and B) In slit lamp examination, a sectorial cataract was seen between 11 and 1 o'clock. C and D) On upward gaze, we found a pale tan tumor that seemed to originate from the superior aspect of the ciliary body and cause a subluxation of the lens. E and F) In ultrasound biomicroscopy (UBM) a partially cystic tumor (arrow) with heterogenous internal reflectivity was revealed, with a protrusion of 5.7 mm and a base of 6.0 mm.

In our examination of the patient, we confirmed that there was a pale tan tumor with fully white apical areas that seemed to originate from the superior aspect of the ciliary body, causing a sectorial-cortical cataract and subluxation of the lens (Fig. 1A to D). A dilated vessel was observed in the overlying conjunctiva. On gonioscopy, the anterior chamber angle was open and lightly pigmented. Ultrasound biomicroscopy (UBM) revealed a partially cystic tumor with heterogenous internal reflectivity, a thickness of 5.7 mm and a largest basal diameter of 6.0 mm (Fig. 1E and F). Scleral transillumination was not performed.

Clinically, the differential diagnosis included a ciliary body medulloepithelioma. The biopsy was considered unsafe due to the potential risk of seeding tumor cells into the orbit. After a discussion of management options with the patient it was agreed that the eye should be enucleated.

**Fig. 2:**
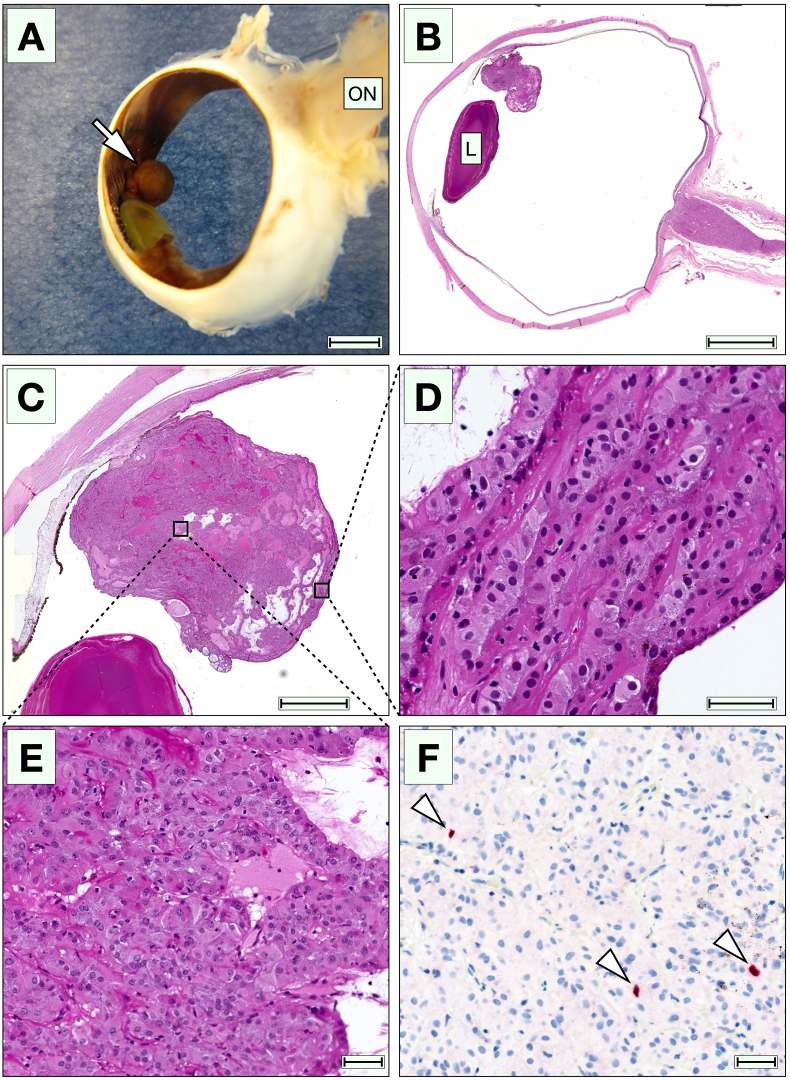
Gross appearance and histopathological examination. A) In gross examination, the lesion had the appearance of a tan ciliary body nodule (arrow). B and C) In low magnification, an endophytic tumor is seen on the inner surface of the ciliary body. The tumor did not infiltrate the iris, anterior chamber angle or the ciliary body itself. D) Various growth patterns were present. Some areas had a trabecular appearance. E) Other areas had a more solid pattern, with the enlarged polygonal cells resting on prominent basement membranes. F) In immunohistochemical staining with the proliferation marker Ki67, up to 2% of the cells were positive (arrowheads) when counting 100 cells in the most positive area. ON, optic nerve. L, lens. Scale bars: A and B, 4 mm. C, 2 mm. D to F, 40 μm.

On gross pathology, a tan, ciliary body nodule was seen with a beige irregular cut surface (Fig. 2A). On histopathological examination, we found a nodular endophytic tumor located on the inner surface of the ciliary body that appeared to arise from the nonpigmented ciliary epithelium (NPCE, Fig. 2B and C). The tumor consisted of enlarged, eosinophilic, polygonal cells, with minor regions of cuboidal and columnar shape, which preserved some morphological characteristics of the NPCE. Their nuclei were round or slightly oval, and some contained a small, decentralized nucleolus. The cells grew in trabecular papillary patterns, with areas of solid and microcystoid growth (Fig. 2D and E). Mitoses were rare, with less than one identified per ten high power fields (×40). Scattered lymphocytes were observed throughout the lesion, with no granulocytes or signs of significant inflammation. Further, we found no necrotic areas, no rosettes, no neural tube-like strands, no photoreceptor differentiation, and no signs of choroidal invasion. A glaucomatous excavation was seen in the optic nerve head.

Some strands of epithelial cells rested on prominent periodic acid-Schiff-positive basement membranes. Immunohistochemically, the tumor was positive for cytokeratin AE1/AE3 (ThermoFisher Scientific, Waltham, MA, USA), Vimentin (Ventana, Oro Valley, AZ, USA), and weakly for S100 (Ventana). HMB45 (Ventana) was negative. The proliferation marker Ki67 (Ventana) was positive in up to 2% of the tumor cells, when counting 100 cells in the most positive area (hot spot, Fig. 2F).

Based on these findings, the diagnosis of NPCEA was established. The patient was referred back to his home clinic and to an ocularist to fit a prosthesis. It was determined that no radiological screening for metastases was required.

## 3 Discussion

In this report, we present a rare case of glaucoma caused by NPCEA, with detection of the tumor only when the development of cataract prompted ocular examination with mydriasis. NPCEA has previously been associated with secondary glaucoma in three patients with neovascularization or anterior uveitis, which may be related to elevated levels of VEGF in vitreous and aqueous humor.^3^^,^^14^^,^^17^ However, our patient had no signs of neither neovascularization nor uveitis, and the relation between his initially elevated IOP and the NPCEA, if any, is unclear. Previous reports have also indicated that NPCEA are typically ultrasonographically solid with a high internal reflectivity, whereas the tumor presented herein was multicystic.^13^

As suggested by Zimmerman in 1970, neoplasms of the NPCE can be divided into congenital and acquired types.^19^ Congenital neoplasms include medulloepithelioma, that arises from the primitive medullary epithelium and is diagnosed at a median age of five years, but also has been diagnosed in adults on very rare occasions.^20^ Acquired neoplasms primarily include adenoma and adenocarcinoma. Zimmerman classified the latter two into solid, papillary, and pleomorphic subtypes, but the tumor presented herein displayed both solid and trabecular papillary patterns. This seems to confirm a previous finding by Shields et al. that most acquired tumors of the NPCE are comprised of a variation of growth patterns.^13^^,^^21^

In this case, the cystic appearance in UBM and the pale appearance in slit lamp examinations required consideration of medulloepithelioma in the differential diagnosis, other possible lesions including amelanotic ciliary body melanoma, granuloma, leiomyoma, and Schwannoma.^12^^,^^13^ In contrast to NPCEA, both malignant medulloepithelioma and melanoma typically require enucleation or plaque brachytherapy.^12^ On histological examination with immunohistochemistry, diagnosis of these entities is straightforward.

Whereas medulloepitheliomas that are confined to the globe confer a 5-year patient survival in excess of 90%, extraocular extension increases the risks dramatically, with a 50 and 40% risk for orbital recurrence and distant metastasis, respectively.^22^, ^23^, ^24^, ^25^, ^26^ Local resections have been used for small medulloepitheliomas occupying less than 3 or 4 clock hours, but have been associated with high rates of local recurrence and secondary enucleation.^24^^,^^25^^,^^27^ In fear of extraocular spread, we are hesitant to recommend a biopsy once the clinical suspicion of a medulloepithelioma is strong enough, and therefore opted for enucleation for the patient described herein.

In conclusion, we report a case of glaucoma secondary to NPCEA, which was detected only after mydriasis. We hope that this report can help raise awareness of this entity among fellow ophthalmologists.

## Statement of ethics

The subject has given his written informed consent to publish his case (including publication of images). All research was conducted ethically in accordance with the Declaration of Helsinki.

## Funding

Support for this study was provided to Gustav Stålhammar from:

• The Swedish Society of Medicine (SLS-971390)
• Region Stockholm (FoUI-981345)
• The Swedish Cancer Society (20 0798 Fk)

The sponsors or funding organizations had no role in the design or conduct of this research.

## Authorship

All authors attest that they meet the current ICMJE criteria.

## Declaration of competing interest

The authors declare that they have no known competing financial interests or personal relationships that could have appeared to influence the work reported in this paper.
