# Supplementary material for: Adenoma of the nonpigmented ciliary epithelium presenting as glaucoma
Source: Am J Ophthalmol Case Rep. 2023 Jun 20;32:101871. doi: 10.1016/j.ajoc.2023.101871 (PMC10319985; doi:10.1016/j.ajoc.2023.101871)
Supplement: Multimedia component 1 [file mmc1.pdf]

Supplementary Material to

Adenoma of the Nonpigmented Ciliary Epithelium Presenting as Glaucoma

Gustav Stålhammar, M.D. Ph.D.[1,2] Maria Fili, M.D. Ph.D.[1,2] Bertil E. Damato, M.D. Ph.D.[1,2]

[1]St. Erik Eye Hospital, Stockholm, Sweden
[2]Department of Clinical Neuroscience, Division of Ophthalmology and Vision, Unit of Ocular Oncology and Pathology, Karolinska Institutet, Stockholm, Sweden

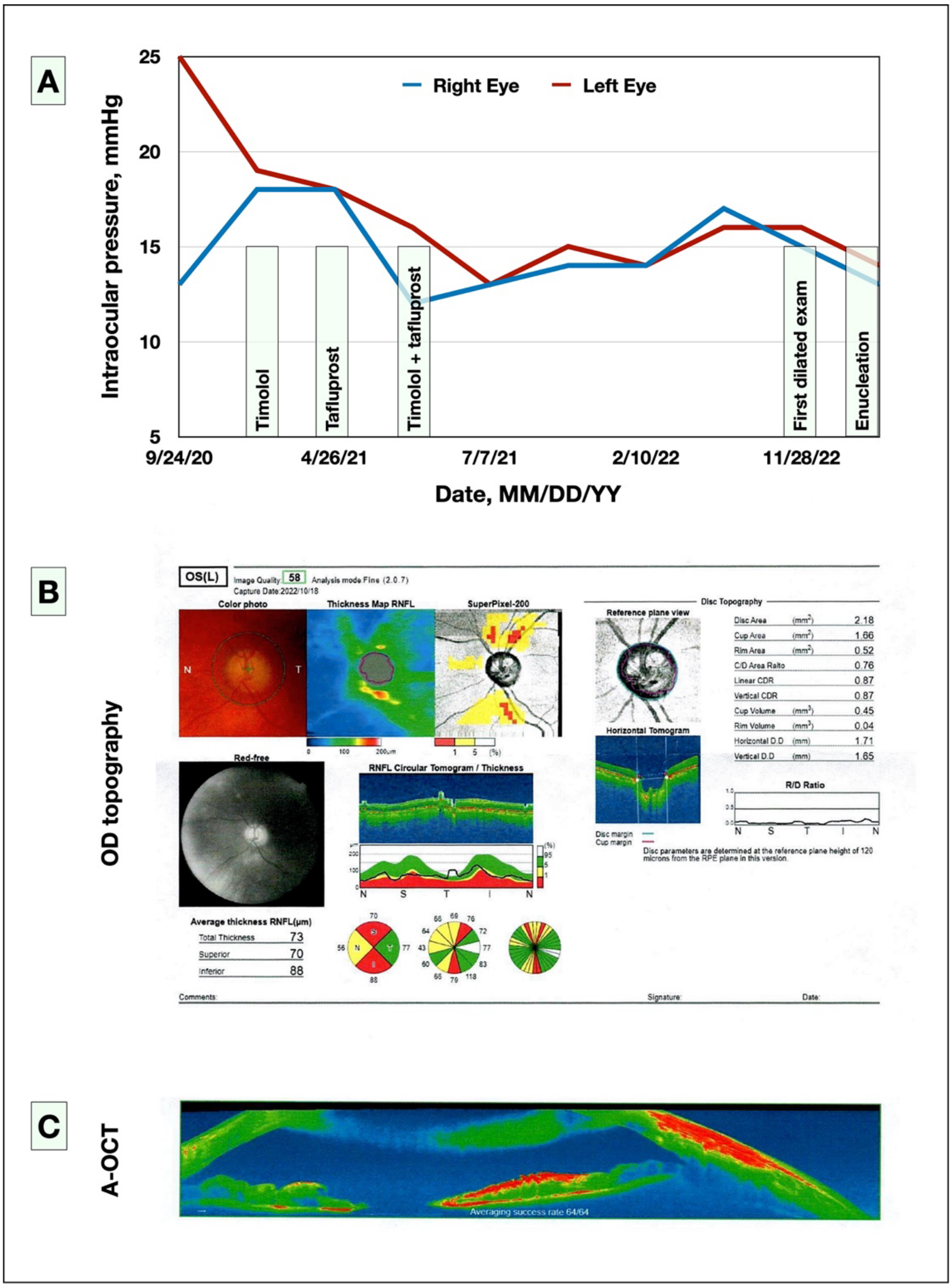

Supplementary figure 1. Intraocular pressures and OCT findings. A) Development of intraocular pressures over time, from presentation at the optometrist in September, 2020, to enucleation in January, 2023. The patient was prescribed timolol, that was later replaced with tafluprost, and then with a combination of timolol and tafluprost. B) Optic disc topography in October, 2022. The retinal nerve fiber layer (RNFL) was thinned in all quadrants, and the cup-disc (C/D) area ratio was 0.76. C) The adenoma of the nonpigmented ciliary body epithelium was not visible on anterior segment OCT. A OCT, anterior segment optical coherence tomography. OD, optic disc.
