# Supplementary material for: Adenoma of the nonpigmented ciliary epithelium presenting as glaucoma
Source: Am J Ophthalmol Case Rep. 2023 Jun 20;32:101871. doi: 10.1016/j.ajoc.2023.101871 (PMC10319985; doi:10.1016/j.ajoc.2023.101871)
Supplement: Multimedia component 2 [file mmc2.pdf]

Consent Form for Identifiable Photographs

Patient's consent for the publication of identifiable photographs in the **AJO**.

Subject of photograph: Roberth Sandtjärn

I give my consent for this photo to appear in the **AJO** and associated publications.

I understand that:

My name will not be published.
The material may be published in the monthly print copy of the **AJO**, which has a circulation of about 12, 000 copies worldwide.
The material may also be placed on the **AJO**'s worldwide website. Both the printed version and the website are seen and read by doctors.

Signed ______________________ Date January 22, 2023

Print Name Roberth Sandtjärn

If you are not the patient, what is your relationship to them?

Witness ______________________ Date ______________
